# The Role of Intestinal Bacteria Overgrowth in Obesity-Related Nonalcoholic Fatty Liver Disease

**DOI:** 10.3390/nu6125583

**Published:** 2014-12-03

**Authors:** Silvia M. Ferolla, Geyza N. A. Armiliato, Cláudia A. Couto, Teresa C. A. Ferrari

**Affiliations:** Departamento de Clínica Médica, Faculdade de Medicina, Universidade Federal de Minas Gerais, Belo Horizonte 30130-100, Brazil; E-Mails: geyzaarmiliato@hotmail.com (G.N.A.A.); clacouto@hotmail.com (C.A.C.)

**Keywords:** fatty liver, nonalcoholic fatty liver disease, nonalcoholic steatohepatitis, small intestinal bacterial overgrowth, gut microbiota, endotoxemia, bacterial translocation

## Abstract

Nonalcoholic fatty liver disease (NAFLD) is the most common chronic liver disease worldwide. It is a progressive disorder involving a spectrum of conditions that include pure steatosis without inflammation, nonalcoholic steatohepatitis (NASH), fibrosis and cirrhosis. The key factor in the pathophysiology of NAFLD is insulin resistance that determines lipid accumulation in the hepatocytes, which may be followed by lipid peroxidation, production of reactive oxygen species and consequent inflammation. Recent studies suggest that the characteristics of the gut microbiota are altered in NAFLD, and also, that small intestinal bacterial overgrowth (SIBO) contributes to the pathogenesis of this condition. This review presents the chief findings from all the controlled studies that evaluated SIBO, gut permeability and endotoxemia in human NAFLD. We also discuss the possible mechanisms involving SIBO, lipid accumulation and development of NASH. The understanding of these mechanisms may allow the development of new targets for NASH treatment in the future.

## 1. Introduction

Currently, nonalcoholic fatty liver disease (NAFLD) is considered the most prevalent chronic liver disease in the western world [1]. It is usually associated with the metabolic syndrome (MS), and encompasses a spectrum of clinicopathological conditions that ranges from simple hepatic steatosis (fatty liver) to hepatic steatosis associated with necroinflammatory lesions (nonalcoholic steatohepatitis (NASH)) with or without hepatic fibrosis that may progress to cirrhosis. The pathogenesis of NAFLD is not fully elucidated. According to the most accepted theory, insulin resistance (IR) is a key factor that initiates hepatic fat accumulation and, potentially, NASH [2,3]. IR affects lipid metabolism as it increases peripheral lipolysis, triglyceride synthesis, and hepatic uptake of free fatty acids (FFA) contributing to the accumulation of triglyceride in the hepatocytes [4]. This excessive deposition of triglyceride in the liver leads to a shift from carbohydrates to FFA mitochondrial beta-oxidation, and may promote lipid peroxidation and accumulation of reactive oxygen species (ROS) in the hepatocytes. These compounds produce a variety of cellular stimuli with subsequent inflammatory response, hepatocellular injury, and fibrosis [2,4].

The liver is constantly exposed to gut microbiota-derived products that activate hepatic toll-like receptor 4 (TLR4), which has been implicated in the development of liver inflammation and fibrosis, and even hepatocellular carcinoma [5,6]. Obese subjects present distinct microbiota composition with relative low proportion of Bacteroidetes and predominance of Firmicutes [7]. This predominance has been associated with a propensity to develop NAFLD features, such as fasting hyperglycemia, hyperinsulinemia, hepatic steatosis, and increased expression of genes involved in *de novo* lipogenesis, independently of the presence of obesity, in animals models [8]. The microbiota composition of humans with NASH also presents lower proportion of Bacteroidetes independently of BMI and dietary fat intake. The low prevalence of Bacteroidetes may facilitate the development of other bacteria phyla that are more efficient in harvesting energy from the diet [9].

NAFLD patients present a high prevalence of small intestine bacterial overgrowth (SIBO) [10,11,12,13,14] and increased gut permeability [13,15] characterized by disruption of the intercellular tight junctions, which is likely to be the underlying mechanism of translocations of bacteria and their products [13]. NASH subjects have elevated plasma levels of LPS associated with a rise in tumor necrosis factor (TNF)-α gene expression in the hepatic tissue, which supports a role of endotoxemia in the development of steatohepatitis [16]. SIBO in NASH individuals is also associated with enhanced hepatic expression of TLR4 and release of interleukin (IL)-8 supporting the hypothesis that SIBO may have an important role in NASH development and progression [14].

The suggested mechanisms to explain the role of SIBO in lipid accumulation and development of NASH are the focus of the present comprehensive review. The understanding of these mechanisms may allow the development of new strategies to prevent or treat NAFLD.

## 2. Relationship between the Gut and the Liver

The human gut microbiota consists of about 10^14^ bacterial cells, including more than 200 species with predominance of anaerobic bacteria [17]. This microbiome contains 100 times more genes than the human genome [18]. At birth, the human gut is sterile, but it is soon colonized by bacteria, whose species are determined by the mode of delivery (vaginal or caesarean section), type of feeding (breast or bottle feed), and introduction of solid food in the diet [19]. The human gastrointestinal tract harbors three dominating bacterial phyla: the gram-positive Firmicutes and Actinobacteria, and the gram-negative Bacteroidetes. The largest bacterial phylum is Firmicutes with 200 genera, such as *Lactobacillus*, *Mycoplasma*, *Bacillus*, and *Clostridium* [20,21]. In adults, almost 60%–80% of the gut microbiota consists of Firmicutes and approximately 20%–40% are Bacteroidetes. The gut microbiota plays several important functions in the host metabolism by the secretion of bioactive metabolites; participates in the development of the intestinal microvilli defense against pathogens by maintaining immunity at the level of the gut; performs the digestion of complex indigestible polysaccharides; synthesizes vitamins; and plays a role in fat storage [19]. The microbiota composition is influenced by diet, age, body weight, infections, medications, intestinal surgeries, and several liver diseases [5,19].

The gut epithelium is a natural barrier that selects entry of useful substances present in the lumen, as nutrients, and keeps at bay bacteria, their bio-products and other potentially harmful elements. Tight junctions, specialized intercellular structures, assist this control. Derangement of the homeostasis between bacteria and the host, as occurs in SIBO (enhanced amount and/or changes in the type of bacteria in the gastrointestinal tract), may cause disruption of the intercellular tight junctions and subsequent increase in intestinal permeability leading to bacterial translocation (BT), *i.e.*, transportation of bacteria and bacterial products from the intestinal lumen into the blood [22].

The portal vein (which drains from the mesenteric veins) and the hepatic artery supply blood to the liver. The portal blood contains products of digestion and also microbial products derived from the gut microbiota. The liver, therefore, consists in the first site of exposure and filtration of microbial products from the gut, such as LPS, lipopeptides, unmethylated DNA, and double-stranded RNA, which may evoke inflammatory reaction contributing to the progression of the liver disorder [5].

It is well known that cirrhosis and other chronic liver diseases favor BT [23], and are also affected by changes in the intestinal microbiota [24,25]. Furthermore, these patients are more likely to develop systemic bacterial infections and complications related to SIBO and increased BT [23,26]. Gut microbiota-derived products activate hepatic TLR4, which has been implicated in the development of alcoholic steatohepatitis, NASH, liver fibrosis and hepatocellular carcinoma [6].

Recent evidence has suggested that gut microbiota may also contribute to the development and progression of liver diseases by modifying the bile acid profile. Bile acids participate in the interaction between the liver and the gut. They are ligands of the farnesoid X receptor (FXR), which is expressed in the liver and gut [27]. The activation of FXR reduces circulating bile acids (feedback mechanism), and participates in the control of the gut-microbiota composition and in the regulation of lipids and glucose homeostasis in the gut-liver axis. All of these factors are involved in the pathogenesis of MS, and hepatic steatosis, inflammation and fibrogenesis [27].

Cirrhotic patients present decreased total fecal bile acids excretion probably by decreasing bile flow. As bile acids have direct bacteriostatic effects, their reduction may favor the development of SIBO [28]. An experimental study demonstrated that the administration of bile acids to cirrhotic mice was followed by normalization of the bile flow and reduction of both SIBO and BT [29]. Experimental models evaluating the relation between NASH and serum concentrations of bile acids have also been developed [30]. Further studies are necessary to clarify this issue.

## 3. Biological and Molecular Basis of SIBO in NAFLD

Obesity characterized by increased body mass index (BMI) or visceral obesity is a well-documented risk factor for NAFLD [1]. Gut microbiota is linked to both: obesity and NAFLD. The microbiota is related to obesity because it can increase energy harvesting from the diet and enhances energy storage. SIBO and increased intestinal permeability are related to NAFLD as they cause endotoxemia with subsequent cytokines release, systemic inflammation and IR [19].

The composition of gut microbiota of obese subjects has been related to less diversity of intestinal bacteria, and altered expression of both bacterial genes and metabolic pathways [7,31]. Ley *et al.* [7] demonstrated that the relative proportion of Bacteroidetes is decreased, whereas the proportion of Firmicutes is increased in obese individuals in comparison with lean people; furthermore, they observed that weight loss is followed by an increase in the proportion of Bacteroidetes. According to a more recent study [32], in obese people, a decrease in Bacteroidetes is accompanied by enhancement in Actinobacteria. The shift in the relative abundance of phyla in obese is associated with increased capacity for harvesting energy from indigestible polysaccharides present in the diet, which are normally broken by glycoside hydrolases and polysaccharide lyases, enzymes that are absent in humans [19,31]. The gut bacteria convert these polysaccharides into monosaccharides and short-chain fatty acids in the colon, which after absorption, lead to triglyceride synthesis in the liver [33].

Furthermore, obese subjects present more H_2_-producing Prevotellaceae and H_2_-utilizing methanogenic Archaea than the normal-weight or post-gastric-bypass individuals. The coexistence of H_2_-producing bacteria with H_2_-utilizing methanogenic Archaea in the gastrointestinal tract of obese persons suggests that H_2_ transfer between bacterial and archaeal species may increase energy uptake by the human large intestine in obese individuals [34].

Evidence suggests that gut microbiota composition can influence the response to a high fatty diet (HFD) and hepatic lipid metabolism, contributing to the development of NAFLD independently of obesity [8]. In an experimental study, in which two mice were fed with a HFD and presented similar body weight gain, one mouse (called responder) developed hyperglycemia and presented high serum concentrations of pro-inflammatory cytokines, whereas the other (called nonresponder), had no alterations in plasma glucose concentrations and presented lower levels of the systemic inflammatory markers. The authors transplanted the gut microbiota from either the responder or the nonresponder mouse to germ free mice. These animals were fed with the same HFD, and also developed comparable obesity. The responder-receiver mice presented fasting hyperglycemia, hyperinsulinemia, hepatic macrovesicular steatosis, high liver concentrations of triglycerides, and increased expression of genes involved in *de novo* lipogenesis. The nonresponder-receiver animals remained normoglycemic and did not develop the other abnormalities. The authors concluded that the gut microbiota determined the propensity to develop NAFLD features. Gut microbiota composition was also different between the responder- and nonresponder-receiver mice: the bacteria phyla associated with the NAFLD-prone and NAFLD-resistant phenotypes were, respectively, Firmicutes and Bacteroides [8].

Another mechanism that explains the role of the gut microbiota in the pathogenesis of NAFLD is the fact that bacteria inhibit gut epithelial expression of fasting-induced adipocyte factor (Fiaf), a suppressor of lipoprotein lipase (LPL). Fiaf is produced not only by the gut, but also by liver and adipose tissue, being an essential regulator of peripheral fat storage [5]. By suppressing Fiaf, the microbiota increases LPL activity in the adipose tissue enhancing the delivery of adipocyte-derived triglycerides [18], which determines storage of triacyglycerols in the liver [17]. Additionally, microbiota is related to IR [31]. In a very interesting human study, the transfer of gut microbiota from lean donors to recipients with MS, via duodenal tube, resulted in increased insulin sensitivity within six weeks [35].

The intestinal microbiota of humans with NASH was studied by Mouzaki *et al.* [9] that found a lower proportion of Bacteroides/Prevotella (herein referred to as Bacteroidetes) in the stool when compared to the individuals with simple steatosis or healthy controls (living liver donors), independently of BMI and dietary fat intake. Without adjusting for BMI, the NASH patients also presented increased number of *Clostridium coccoides* in the stool in comparison to the individuals with simple steatosis. According to the authors, the low prevalence of Bacteroidetes may facilitate the development of other bacteria phyla that are more efficient in harvest energy from the diet.

Zhu *et al.* [36] also characterized the gut microbiomes in NASH subjects. According to their findings, there are increased abundance of alcohol-producing *Escherichia* in the microbiota of these patients as well as elevated blood-ethanol concentrations leading to increased oxidative stress and liver inflammation due to alcohol metabolism. Indeed, in addition to the increased production of ethanol, the intestinal microbiota also synthesizes LPS that promotes release of the pro-inflammatory cytokine TNF-α and IL-6 from the hepatic macrophages, which contributes to liver damage, disrupts normal hepatocyte function, leads to mitochondrial oxidative stress, and reduces the clearance of toxins by the hepatocytes [37]. Corroborating these findings, Ruiz *et al.* [16] demonstrated that fatty liver patients presented elevated plasma levels of LPS, which were further increased in the individuals with NASH. The high LPS levels were also associated with a rise in TNF-α gene expression in the hepatic tissue.

In humans, SIBO has been associated with endogenous ethanol production [38], which contributes to the functional and morphological damages to the small bowel, increasing its permeability to endotoxins derived from the intestinal lumen [13]. SIBO is described in NASH patients [10,11,12,13,14] and is associated with enhanced hepatic expression of TLR4 and release of interleukin IL-8 [14]. These findings support the hypothesis that SIBO may have an important role in NASH development and progression by inducing proinflammatory signaling cascades.

**Figure 1:**
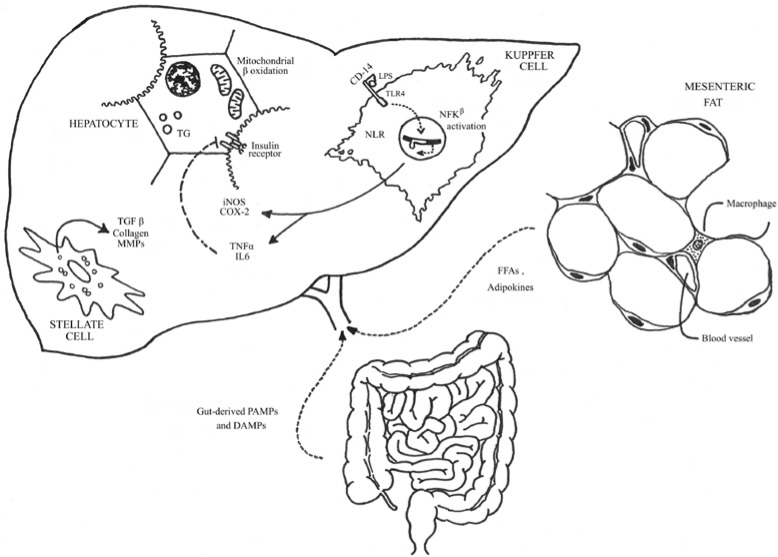
Alterations in gut microbiota increase intestinal permeability favoring the absorption of pathogen-associated molecular patterns, such as lipopolysaccharide (LPS). This phenomenon activates the TRL4 receptors that increase the NF-κB-related gene transcription in the Kupffer cells triggering inflammatory pathways by the activation of proinflammatory genes, such as TNF-α, IL-6, IL-8, and IL-12 genes, and by generating reactive oxygen species (ROS). The consequent inflammatory response induces production of profibrotic factors by the hepatic stellate cells; impairs insulin signaling with a subsequent increase in FFAs afflux; and alters mitochondrial beta-oxidation, which results in hepatic steatosis and inflammation. PAMPs: pathogen-associated molecular patterns; DAMPs: damage associated molecular patterns; TGF-β: transforming growth factor-β; MMPs: metalloproteinases; LPS: lipopolysaccharide; NF-κB, nuclear factor-κB; TLR, toll-like receptor; ROS, reactive oxygen species; iNOS, inducible nitric oxide synthase; TNF-α: tumor necrosis factor-α; IL-6: interleukin-6; COX-2: cyclooxygenase-2; TG: triglycerides; FFAs, free fatty acids (Adapted from Meli R, Raso GM, Calignano A. Role of innate immune response in non-alcoholic Fatty liver disease: metabolic complications and therapeutic tools [41].)

Several mechanisms have been suggested to explain the association between SIBO and fat accumulation in the hepatocytes. It is well known that inflammation plays an important role in the development and progression of the hepatic damage and fibrosis in chronic liver disorders [39]. The liver contains important components of the immune system, including macrophages, dendritic cells and natural killer T cells, which act as first-line defense against microorganisms and endotoxin. TLRs present on the innate immune cells consist of a family of type I transmembrane proteins that recognize pathogen-associated molecular patterns (PAMPs) and damage associated molecular patterns (DAMPs) present on endogenous ligands, and initiate an adaptive immune response signaling cascade leading to activation of proinflammatory genes, such as TNF-α IL-6, IL-8, and IL-12 genes. LPS, a component of the gram-negative bacteria cell membrane and the active component of endotoxin is the most studied PAMP. The liver is frequently exposed to these PAMPs due to BT. These molecules leave the gut and reach the liver through the portal vein [40]. LPS binds to LPS-binding protein, which in turn, binds to CD14 and activates TLR4 in the Kupffer cells triggering an essential inflammatory cascade, which includes stress-activated and mitogen-activated protein kinases, Jun N-terminal kinase (JNK), p38, interferon regulatory factor 3, and the nuclear factor (NF)-κB pathway [19] (Figure 1).

All types of liver cells, including hepatocytes, Kupffer cells, sinusoid endothelial cells, hepatic stellate cells, biliary epithelial cells, as well as immune cells have a wide expression of TLRs [6,42]. The liver is constantly exposed to TLR ligands. TLRs binding to the corresponding ligands induces potent inflammatory cascade as a result of the activation of NF-κB, production of proinflammatory cytokines, and activation of c-JNK [6,43]. The synthesis of proinflammatory cytokines leads to prolonged inflammation and hepatocyte damage [40]. Indeed, the TLRs, other sensors of PAMPs and DAMPs, are the inflammasomes, which are formed by a molecular macrocomplex that includes the enzyme caspase-1, whose activation causes the release of bioactive IL-1β and/or IL-18 [22]. Recent evidence suggests that inflammasome is involved in NAFLD/NASH progression via modulation of the gut microbiota [44]. Genetic inflammasome deficiency associated with dysbiosis determines increased concentration of bacterial products in the portal blood which may exacerbate steatosis and increase TNF-α expression [44].

The activation of inflammatory pathways also causes impairment of the insulin signaling, with decreased phosphorylation of the insulin receptor, insulin receptor substrate (IRS) and Akt, as well as increased inhibitory serine phosphorylation of IRS-1 [31].

It has been demonstrated in animal models that a four-week HFD increases LPS contained in the gut microbiota and plasma LPS concentrations two to three times, which is considered metabolic endotoxemia. The induction of metabolic endotoxemia in mice, by continuous subcutaneous infusion of LPS during four weeks, was followed by a rise in the following parameters: fasting glycemia, insulinemia, markers of inflammation, liver triglyceride content, liver insulin resistance, and whole body, liver and adipose tissue weight gain in a similar amount as occurred in HFD fed mice [45]. Large amount of fructose consumption is also related to increase in endotoxin serum levels, proinflammatory response and steatosis. It was demonstrated in an elegant study conducted by Bergheim *et al.* [46] that mice fed with fructose showed increased endotoxin levels in the portal blood, and higher intrahepatic lipid accumulation, lipid peroxidation and TNF-α expression.

TNF-α is a well-known cytokine related to the progression of NAFLD; and sterol regulatory element**-**binding transcription factor 1 (SREBP-1c) is a factor involved in lipogenesis [45,47,48]. Postic *et al.* [49] demonstrated that expression of TNF-α stimulates the expression of SREBP-1c. In a very recent study, Fukunishi *et al.* [50] demonstrated that the administration of LPS to rats increased hepatic TNF-α and SREBP-1c expressions, suggesting the possibility that LPS may play a significant role in the progression of hepatic steatosis. The rats that received LPS also presented higher expression of fatty acid synthase, acetyl-CoA carboxylase, ATP-citrate lyase, medium-chain acyl-CoA dehydrogenase, and long-chain acyl-CoA dehydrogenase, which are enzymes involved in the lipogenetic pathway. These results suggest that LPS could be involved in mitochondrial fatty acid β-oxidation. The plasma levels of adiponectin, which is a cytokine that protects the liver, were decreased in these animals; therefore, it is reasonable to conclude that LPS may affect the adipocytes reducing adiponectin secretion, and then, contributing to liver damage [50].

Since the 1970s, some publications have suggested that SIBO could play a role in the pathogenesis of NAFLD. Descriptions of fatty degeneration have been reported in morbidly obese patients with jejuno-ileal bypass [51,52] and in small bowel diverticulosis [53], both conditions favoring bacterial overgrowth. SIBO has been defined as a total bacteria growth of more than 105 colony-forming units per milliliter of intestinal fluid [12]. Although bacteriological analysis of jejune aspirate is the most accurate procedure for confirming the presence of SIBO [54], because of the constraints (namely, the high cost and the discomfort caused by the test) in obtaining cultures, different breath tests, including the glucose breath test (GBT), have been used as surrogate methods [12].

Most controlled trials demonstrated that the prevalence of SIBO in NAFLD ranges from 50% to 77.8% [10,11,13,14]. Volynets *el al.* [15] did not find any differences in the prevalence of SIBO when they compared NAFLD patients and healthy subjects; however, they observed higher levels of blood alcohol and presence of endotoxin in the plasma of the NAFLD individuals, which suggest synthesis of ethanol by the gut microbiota and increased intestinal permeability to bacterial endotoxin, respectively.

The differences in SIBO prevalence among the studies [10,11,13,14,15] might have been influenced by the variation of the substrates employed in the hydrogen breath tests used to diagnose SIBO (GBT, combination of the 14C-D-xylose and lactulose breath test (LBT) or LBT). No studies were carried out measuring hydrogen and methane in the breath to ensure that there were no false negatives due to the presence of gastrointestinal bacteria not producing hydrogen [55].

Indeed, the differences in SIBO prevalence among the studies may also have been influenced by the heterogeneity of the study populations, such as different proportions between NASH and fatty liver patients; differences in life style; differences in ethnicities; and differences in the prevalence of diabetes. Several authors emphasize the importance of diabetes or glucose intolerance in the development of SIBO [10,11,13,14,15]. Hyperglycemia causes autonomic neuropathy, which in turn, leads to slow gastric emptying and decreased intestinal motility [45,47]. The impaired contractile activity of the stomach may cause retention of indigestible material in the gastric lumen favoring the development of SIBO [56]. Likewise, the delayed intestinal transit time facilitates retrograde colonization of the small bowel by colonic bacteria and subsequent SIBO. However, SIBO [12] and delayed orocecal transit time [57,58] have been observed in NAFLD patients even in the absence of diabetes or impaired glucose tolerance. Sajjad *et al.* [11] observed lower plasma levels of ghrelin in NASH subjects; as ghrelin has a prokinetic effect similar to motilin, the authors speculated that the low ghrelin concentrations could be related to the occurrence of SIBO.

SIBO has been independently associated with the severity of hepatic steatosis on liver histology [12,13]. As liver biopsy is not always available, markers of hepatic damage, such as plasminogen activator inhibitor 1 (PAI-1), have been used in some studies [15,59]. The liver seems to be involved in plasma PAI-1 regulation and its circulating levels are increased in the presence of IR, central obesity, and elevated serum concentrations of hepatic enzymes, which are common features of NASH. Recent studies have demonstrated that increased serum levels of PAI-1 are associated with steatosis, hepatic fibrosis [60,61,62], and increased serum levels of endotoxin [15,59].

SIBO may enhance intestinal permeability favoring endotoxemia [58], and thus, oxidative stress in the hepatocytes. Miele *et al.* [13] demonstrated a high prevalence of SIBO, increased intestinal permeability (urinary excretion of 51Cr-ethylene diamine tetraacetate (51Cr-EDTA) test), and disruption of the intercellular tight junctions of the gut (immunohistochemical analysis of zona occludens-1 (ZO-1) expression in duodenal biopsy specimens) in NAFLD patients. Furthermore, the increased gut permeability was correlated with the severity of the hepatic steatosis. Volynets *et al.* [15] using the lactulose-mannitol test also identified increased intestinal permeability in NAFLD patients whereas Wigg *et al.* [10] did not find any differences in gut permeability between NAFLD subjects and controls using the lactulose and rhamnose test. It is noteworthy that different protocols, substrates and techniques were employed it the studies evaluating intestinal permeability in NAFLD; therefore, these findings should be interpreted with caution.

Other authors, using plasma endotoxin concentrations and TLR4 expression (endotoxin receptor) as markers of intestinal permeability, found similar results [14,19,58,59]. According to their findings, NAFLD patients present higher serum levels of the endotoxin core antibodies EndoCAb IgG (marker of endotoxin exposure) [58], higher levels of endotoxin [15,59], and increased expression of TLR4 on the liver [59] and on CD14^+^ cells [14].

Fat and fructose consumption was demonstrated to be related to gut microbiota and endotoxin serum levels in rodent models of NAFLD and in patients with this disorder [45,46,59,63,64]. Dietary fructose intake is associated with increased intestinal translocation of endotoxin and increased serum levels of PAI-1, which may contribute to the development of NAFLD in humans [59]. Recently, Volynets *et al.* [15] also observed that carbohydrate intake correlated positively with PAI-1, endotoxin and alanine aminotransferase (ALT) plasma levels in NAFLD patients. One year late, the same group of authors [65] observed that the reduction of fructose intake (reduction of 50% in comparison with baseline) during six months was associated with a decrease in hepatic lipid content, BMI, fasting plasma insulin concentrations, and serum levels of the aminotransferases, endotoxin and PAI-1.

Walker *et al.* [66], investigated in a group of 37 obese young adults the presence of fructose malabsorption, assessed by hydrogen breath test, and correlated it with the grade of steatosis measured by magnetic resonance imaging. The patients exhibited high consumption of fructose-containing beverages. The authors observed a negative correlation between fructose malabsorption and grade of steatosis, suggesting that fructose malabsorption might be protective against fatty liver disease. The association between dietary intakes, SIBO and intestinal permeability in NAFLD patients is still an area for further evaluation.

A summary of the clinical trials in which the presence of SIBO in NAFLD patients was investigated using breathing tests are presented in Table 1.

## 4. Treatment of SIBO in NAFLD Patients

Early studies with antibiotic therapy have shown contradictory effects on liver damage related to SIBO [11,57,67]. Although oral ciprofloxacin, for five days, have been effective in treating SIBO in all but one patient with NASH, the authors observed increased fasting insulin levels after the use of the antibiotic. This last finding was contrary to what was expected since a reduction in bacteria activity should cause a decrease in both inflammation and IR [11]. Norfloxacin treatment during two weeks had no effects on ALT levels, LBT, or EndoCAb titers in patients with NAFLD [57]. In an experimental study, Wu *et al.* [67] investigated the effect of cidomycin on NASH related to SIBO. The treatment was followed by significant decrease in the serum levels of ALT, aspartate aminotransferase (AST) and TNF-α in the NASH rats. More studies with antibiotic in NAFLD/NASH patients are necessary before suggesting their rational use to treat this condition.

Probiotics are defined as live microorganisms that when consumed in adequate amounts confer a healthy benefit to the host [5]. Nowadays, they have been considered a promising treatment modality of NAFLD as they modulate gut microbiota, modify the gut barrier function, and have immunomodulatory, anti-inflammatory and metabolic effects [5]. Various interventional studies (Table 2) [37,68,69,70,71,72,73] on the use of oral probiotics to modify gut microbiota in NAFLD patients have demonstrated improvement of the inflammatory parameters, oxidative stress markers and liver biochemistry. According to the results of a recent meta-analyses, probiotic therapy is able to reduce liver aminotransferases, total-cholesterol, TNF-α levels and IR in NAFLD individuals suggesting that modulation of the gut microbiota represents a new complementary therapeutic approach in NAFLD [74]. However, it is important to emphasize that the studies differ regarding the probiotic doses, strains of bacteria and duration of treatment, which hamper the establishment of the best intervention [22].

**Table 1:** Summary of the clinical trials evaluating SIBO, gut permeability and endotoxemia in human NAFLD.

Study	Hepatic Disorder and Sample Size	Variables	Results
Wigg *et al.*, 2001 [10]	22 NASH (23% DM) *vs.* 23 controls (4% DM)	SIBO, gut permeability, endotoxin, TNF-α	NASH group: higher prevalence of SIBO (50% *vs.* 22%; *p* = 0.048); higher mean TNF-α levels (*p* = 0.01).
Sajjad *et al.*, 2005 [11]	12 NASH (41.6% DM) *vs.* 11 healthy controls	SIBO, ghrelin, insulin, ethanol	NASH group: higher prevalence of SIBO (50% *vs.* 9.1%; *p* = 0.025; lower plasma levels of acylated ghrelin (*p* = 0.015); higher fasting insulin concentrations (*p* < 0.006).
Soza *et al.*, 2005 [57]	10 nondiabetic NAFLD *vs.* 10 healthy controls	OCTT, EndoCAb IgG, IgM	NAFLD group: higher basal breathed H2 (*p* = 0.0084); prolonged OCTT (*p* = 0.0037).
Fu *et al.*, 2006 [58]	10 nondiabetic NASH *vs.* 10 healthy controls	OCTT, EndoCAb IgG	NASH group: prolonged OCTT (*p* = 0.00032); higher EndoCAb IgG titers (*p* = 0.011).
Sabaté *et al.*, 2008 [12]	146 morbidly obese referred for bariatric surgery *vs.* 40 healthy controls	SIBO, liver biopsy	Obese group: higher prevalence of SIBO (17.1% *vs.* 2.5%; *p* = 0.031). SIBO (*p* = 0.005) and MS (*p* = 0.006) were independently associated with severe hepatic steatosis.
Thuy *et al.*, 2008 [59]	12 nondiabetic NAFLD and 6 healthy controls	Diet, endotoxin, TLR4, PAI-1 plasma and liver	NAFLD group: consumed more fructose (*p <* 0.05); higher plasma levels of endotoxin (*p <* 0.05), PAI-1(*p <* 0.05), hepatic TLR4 (*p <* 0.05) and PAI-1 mRNA expression (*p <* 0.05). PAI-1 concentrations correlated with endotoxin levels (*r* = 0.83; *p <* 0.005) and with hepatic TLR4 mRNA expression (*r* = 0.54; *p <* 0.05). Hepatic mRNA expression of PAI-1 correlated with dietary intakes of carbohydrates (*r* = 0.67; *p <* 0.01), fructose (*r* = 0.58; *p <* 0.01), glucose (*r* = 0.58; *p <* 0.01) and sucrose (*r* = 0.70; *p <* 0.01).
Miele *et al.*, 2009 [13]	35 NAFLD (34% MS) *vs.* 27 untreated celiac disease (14.5% MS) *vs.* 24 healthy controls	SIBO, gut permeability, tight junctions, liver biopsy	NAFLD group: higher prevalence of SIBO (60% *vs.* 20.8%; *p <* 0.001), higher gut permeability (*p <* 0.001). SIBO and gut permeability correlated with the severity of steatosis (*p <* 0.001 and *p <* 0.05, respectively).
Shanab *et al.*, 2011 [14]	18 NASH (33%DM) *vs.* 16 healthy controls	SIBO, LBP, TLR2 and 4 on CD14+ cells, IL-1β, IL-6, IL-8, and TNF-α	NASH group: higher prevalence of SIBO (77.78% *vs.* 31.25%; *p <* 0.0001), higher TLR4 on CD14+ cells expression (*p <* 0.05); higher levels of IL-8 (*p* = 0.04), which correlated positively with TLR4 expression (*r* = 0.5123, *p* = 0.036).
Volynets *et al.*, 2012 [15]	20 NAFLD (25% pre-diabetic) *vs.* 10 healthy controls	Diet, SIBO, OCTT, gut permeability, blood alcohol, endotoxin, PAI-1	NAFLD group: higher gut permeability, blood alcohol and endotoxin levels (for all, *p <* 0.05). Consumed more energy, carbohydrate, fructose, sucrose (for all, *p <* 0.05) and more glucose, protein and animal-derived protein (for all, *p <* 0.01).

SIBO: small intestinal bacterial overgrowth; NAFLD: nonalcoholic fat liver disease; NASH: nonalcoholic steatohepatitis; DM: diabetes mellitus; TNF-α: tumor necrosis factor alpha; OCTT: orocecal transit time; EndoCAb IgG, IgM: IgG and IgM endotoxin core antibodies; TLR4: toll-like receptor 4; PAI-1: plasminogen activator inhibitor 1; MS: metabolic syndrome; LBP: LPS binding protein.

**Table 2:** Summary of clinical trials using probiotics to treat NAFLD.

Study	Hepatic Disorder and Sample Size	Intervention	Outcome
Loguercio *et al.*, 2002 [68]	10 NASH; 12 chronic HCV infection; 10 alcoholic cirrhosis	Mixture of *Lactobacillus and Bifidobacterium* + FOS + vitamins and minerals for 2 months	NASH patients: decrease in ALT, GGT, MDA, 4-HNE, TNF-α levels.
Loguercio *et al.*, 2005 [69]	22 NAFLD; 20 alcoholic cirrhosis; 20 HCV-related chronic hepatitis; 16 HCV-related cirrhosis	VSL#3 formula for 3 months	NAFLD and alcoholic cirrhosis groups: decrease in MDA, 4-HNE levels. All groups: decrease in S-NO levels.
Vajro *et al.*, 2011 [70]	20 NAFLD children (10 probiotic; 10 placebo)	*L. rhamnosus* for 8 weeks	Experimental group: decrease in ALT, antipeptidoglycan-polysaccharide antibodies levels.
Aller *et al.*, 2011 [71]	28 NAFLD (14 probiotic; 14 placebo)	*L. bulgaricus* and *Streptococcus thermophiles* for 3 months	Experimental group: decrease in AST, ALT, GGT levels.
Malaguarnera *et al.*, 2012 [37]	66 NAFLD (34 probiotic; 32 placebo)	*B. longum* + FOS for 24 weeks	Experimental group: decrease in TNF-α, CRP, AST, HOMA-IR, endotoxin levels; steatosis; NASH activity index.
Wong VW *et al.*, 2013 [72]	20 NAFLD (10 probiotic; 10 placebo)	Lepicol probiotic formula	Experimental group: decrease in AST levels; IHTG.
Eslamparast *et al.*, 2014 [73]	52 NAFLD (26 synbiotic; 26 placebo)	Synbiotic formula for 28 weeks	Experimental group: decrease in ALT, AST, GGT, CRP, TNF-α, nuclear factor κ-B levels; improvement fibrosis score.

NAFLD: nonalcoholic fatty liver disease; NASH: nonalcoholic steatohepatitis; HCV: hepatitis C virus; ALT: alanine aminotransferase; GGT: gamma-glutamyl transpeptidase; MDA: malondialdehyde; 4-HNE: 4-hydroxynonenal; TNF-α: tumor necrosis fac tor; FOS: fructooligosaccharides; VSL#3: mixture of probiotics; S-NO: *s*-nitrosothiols; CFU: colony-forming unit ; BMI: body mass index; AST: aspartate aminotransferases; CRP: C-reactive protein; HOMA-IR: homeostasis model assessment of insulin resistance; IHTG: intrahepatic triglycerides.

## 5. Conclusions

The data described here support the notion that SIBO induces an immune imbalance leading to a state of chronic inflammation, mitochondrial dysfunction, hepatic fat accumulation and NASH. More studies in humans are necessary to better understand the cell-specific recognition and intracellular signaling events involved in recognizing gut-derived microbes and to set up how achieve an optimal balance in the gut-liver axis in order to improve NAFLD.
